# Nanomaterial-Powered Biosensors: A Cutting-Edge Review of Their Versatile Applications

**DOI:** 10.3390/mi16091042

**Published:** 2025-09-11

**Authors:** Payal Patial, Manish Deshwal, Shonak Bansal, Anjana Sharma, Kamaldeep Kaur, Krishna Prakash

**Affiliations:** 1 Department of Electronics and Communication Engineering, University Institute of Engineering, Chandigarh University, Mohali 140413, Punjab, India; deshwal.manish@gmail.com; 2 Department of Physics, University Institute of Sciences, Chandigarh University, Mohali 140413, Punjab, India; anjana87sharma@gmail.com (A.S.); kamalbillingz@gmail.com (K.K.); 3 Department of Electronics and Communication Engineering, NRI Institute of Technology, Agiripalli, Eluru 521212, Andhra Pradesh, India; k_krishna2k7@yahoo.co.in

**Keywords:** biosensor, carbon nanotubes, nanowires, nanorods, quantum dots

## Abstract

Optimal sensing devices exhibit a combination of key performance attributes, including an extensive detection limit, exceptional selectivity, high sensitivity, consistent repeatability, precise measurement, and rapid response times with efficient analyte flow. In recent years, biosensing platforms incorporating nanoscale materials have garnered considerable attention due to their diverse applications across various scientific and technological domains. The integration of nanoparticles (NPs) in biosensor design primarily bridges the dimensional gap between the signal transduction element and the biological recognition component, both of which operate at nanometer scales. The synergistic combination of NPs with electrochemical techniques has facilitated the development of biosensors characterized by enhanced sensitivity and superior analyte discrimination capabilities. This comprehensive analysis examines the evolution and recent advancements in nanomaterial (NM)-based biosensors, encompassing an extensive array of nanostructures. These consists of one-dimensional nanostructures including carbon nanotubes (CNTs), nanowires (NWs), nanorods (NRs), and quantum dots (QDs), as well as noble metal and metal and metal oxide nanoparticles (NPs). The article examines how advancements in biosensing techniques across a range of applications have been fueled by the growth of nanotechnology. Researchers have significantly improved biosensor performance parameters by utilizing the distinct physiochemical properties of these NMs. The developments have increased the potential uses of nanobiosensors in a wide range of fields, from food safety and biodefense to medical diagnostics and environmental monitoring. The continuous developments in NM-based biosensors are the result of the integration of several scientific areas, such as analytical chemistry, materials science, and biotechnology. This interdisciplinary approach continues to drive innovations in sensor design, signal amplification strategies, and data analysis techniques, ultimately leading to more sophisticated and capable biosensing platforms. As the field progresses, challenges related to the scalability, reproducibility, and long-term stability of nanobiosensors are being addressed through innovative fabrication methods and surface modification techniques. These efforts aim to translate the promising results observed in laboratory settings into practical, commercially viable biosensing devices that can address real-world analytical challenges across various sectors.

## 1. Introduction

Biosensors are analytical devices that integrate biological recognition elements with signal transduction and amplification mechanisms, and they have become crucial tools for monitoring biomarkers and early disease detection. The timely identification of pathological conditions significantly enhances treatment efficacy and patient outcomes, underscoring the critical need for advanced sensing platforms capable of detecting problematic organic and inorganic biomolecules in living systems [1]. The genesis of biosensors can be traced back to 1956, when American biochemist Leland C. Clark Jr. developed a device to measure blood oxygen levels using what became known as the Clark electrode [2]. Cammann later came up with the name “biosensor” in 1977, defining these devices as analytical instruments combining bioreceptors (such as cells, antibodies, DNA, or enzymes), transducers (for signal conversion), and amplifiers (for signal processing and enhancement) [1].

The evolution of biosensor technology is typically categorized into five distinct generations. First-generation biosensors measured both analyte composition and bioreceptor reaction products to generate a response signal. Clark’s pioneering work laid the foundation for this generation, which saw significant advancements, including the development of amperometric enzyme electrodes for the detection of glucose in 1962 by Clark [3], the first enzyme electrode by Updike and Hicks in 1967 [4], potentiometric urea sensors by Guilbault and Montalvo in 1969 [5], and optical biosensors for alcohol detection by Lubbers and Opitz in 1975 [6].

Second-generation biosensors enhanced analytical performance through the incorporation of auxiliary co-reactants and enzymes, giving rise to amperometric biosensors acting as mediators [7]. The third, fourth, and fifth generations marked a paradigm shift, with bioreceptors becoming integral components of the sensing element. These advanced platforms established direct electron transferring interfaces between electrodes and receptors, eliminating the requirement for intermediaries. This design approach offered advantages such as cost-effectiveness, reproducibility, and heightened sensitivity [8].

**Figure 1:**
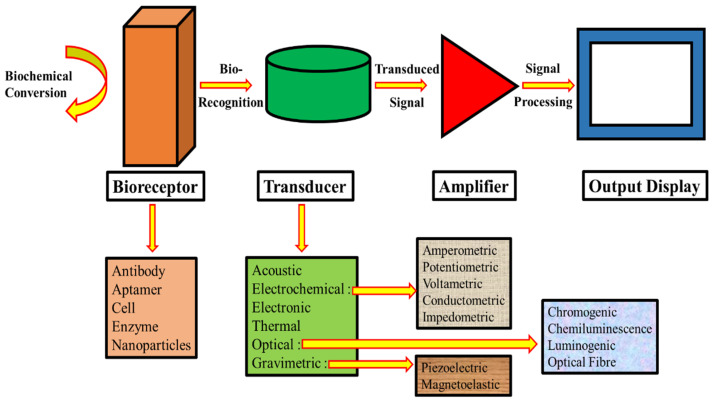
Biosensor essentials: bioreceptor → transducer → amplifier—enabling recognition, conversion, and signal enhancement for accurate detection.

**Figure 2:**
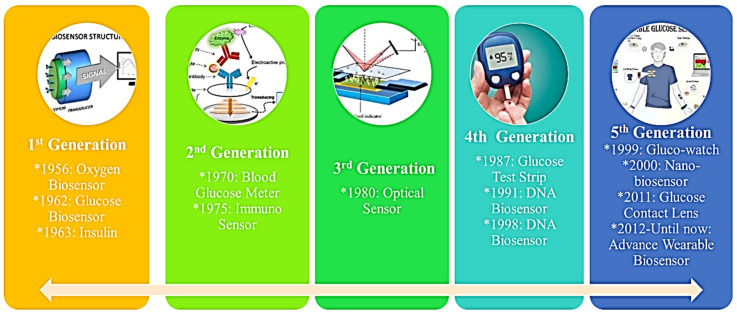
From the enzyme electrode of Clark’s glucose sensor to today’s flexible, non-invasive wearables—tracing the key milestones in biosensor evolution.

The structural framework of a biosensor comprises the following three fundamental elements: the bioreceptor interacting with the target analyte, the transducer converting the biological interaction into a measurable signal, and the amplifier that enhances and processes the generated signal for the interpretation. Figure 1 illustrates a simplified model of the basic parts of a biosensor, emphasizing the importance of the amplifier, transducer, and bioreceptor. This modular architecture allows the development of highly sensitive and specific analytical devices for a wide range of applications. The field of biosensors has witnessed numerous milestones that have propelled its advancement. These achievements include the diversification of bioreceptor types, the integration of NMs for enhanced performance, the devising of label-free detection methods, and the incorporation of advanced signal processing algorithms. Additionally, the integration with microfluidic systems and the miniaturization of biosensing platforms have expanded their applicability in point-of-care diagnosis and field-deployable sensing solutions. As research in biosensor technology continues to progress, emerging trends focus on enhancing selectivity, improving long-term stability, and developing multiplexed sensing capabilities. Figure 2 represents the convergence of biosensors with other cutting-edge approaches, such as wireless communication and Artificial Intelligence (AI), which promise to further revolutionize their utility in environmental monitoring, healthcare, and various other sectors that require rapid, accurate, and on-site analytical capabilities.

### Features and Categories of Biological Sensing Systems

The engineered biosensor prototype must exhibit specific attributes to yield optimal outcomes, thereby advancing societal health and well-being. Key characteristics include the following:

1. Detection Limit and Response Linearity: Contemporary applications demand biosensors with exceptional sensitivity. Environmental monitoring requires detection capabilities in the parts-per-million range, while medical diagnostics often necessitate sensitivities from nanograms to femtograms per milliliter. Furthermore, the linear response of the device across varying analyte concentrations is essential for quantitative accuracy [9].
2. Durability: The long-term stability of a biosensor is a crucial determinant of its commercial viability. A major challenge is signal attenuation with time, which calls for careful consideration throughout the design stage. Interestingly, the rate of deterioration is directly proportional to the temperature and increases at higher temperatures [10].
3. Replicability: The capacity of a biosensor to produce consistent findings over several trials is a crucial performance indicator because of their dependability; devices that exhibit great replicability are exceptionally sought after. The biosensor’s overall reliability is enhanced by its capacity to replicate with great accuracy and precision, which make it a useful analytical tool [9].
4. Specificity: The ability of a biosensor to discriminate between molecules is the most important factor in its design. The device must reliably identify the target analyte within a heterogeneous matrix containing structurally similar compounds or potential interferents. This selectivity is the cornerstone of biosensor functionality, ensuring accurate detection in complex biological or environmental samples [10].

**Figure 3:**
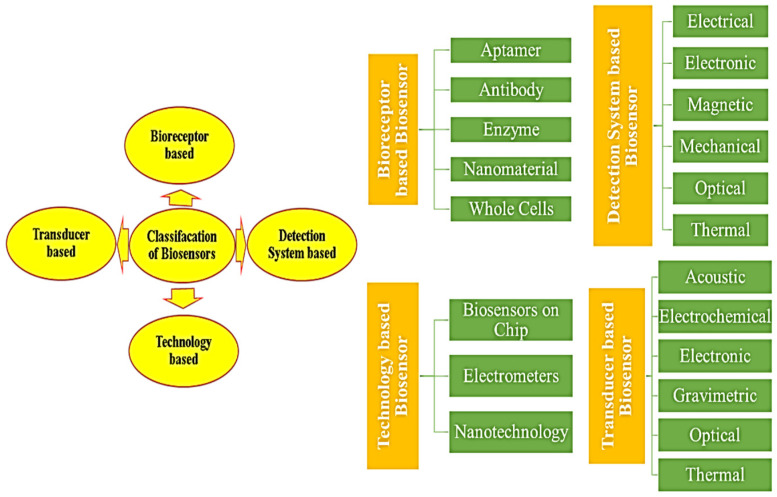
Diverse biosensor classifications by detection target, transduction method, recognition element, and technological platform.

Figure 3 provides a thorough taxonomy of biosensors, divided into groups according to transducer technology, bioreceptor kinds, and detection processes. This classification scheme provides valuable insights for researchers, facilitating the selection of optimal biosensor designs for specific applications.

The categorization of biosensors can be delineated depending on four primary criteria, outlined as follows:

1. Bioreceptor type utilized in device construction.
2. Transducer mechanism employed.
3. Underlying technology driving the device design.
4. Detection system implemented.

This review will primarily focus on NM-based biosensors, given their increasing significance and broad applicability across various fields.

## 2. Development of NP-Based Biosensors and Nanotechnology

**Figure 4:**
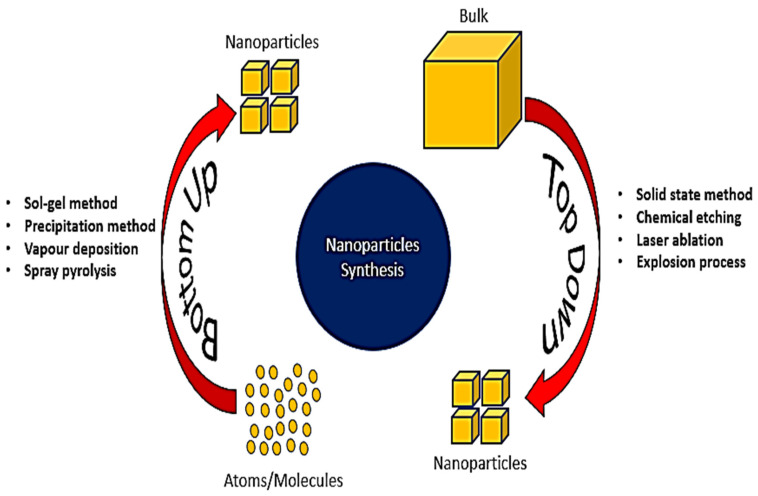
Top-down vs. bottom-up nanomaterial synthesis: from bulk breakdown to atom-by-atom construction.

To fulfill the high demand for biosensors in almost every scientific and technological sector, scientists investigate novel materials at the nanoscale level that may be used to improve sensor technology. Opioids are drugs that have a combination of medicinal advantages and possible risks; they are commonly used as powerful painkillers for controlling pain. Overdose incidents and the potential for addiction-related actions highlight the importance of meticulous tracking. The worldwide growth in the intake and misuse of illegal drugs requires accurate and effective methods of monitoring. Within this framework, the incorporation of efficient nanostructures into biosensor systems represents a viable approach to drug identification, facilitating prompt and precise detection. Saman Sargazi et al. have published a study that explores the subject of opioid-specific nanobiosensors and provides a thorough overview of this emerging field [11,12]. This article carefully examines the field of NMs and provides an example of how they might be used as opioid-specific biosensing instruments. Attention is also given to the molecules that are being studied and the corresponding detection limits, which together influence the accuracy and spectrum of the nanobiosensor devices. Nanotechnology has made significant strides in both growth and utilization during the past two to three decades [13]. Different nanoparticles (NPs) have been developed and are employed to improve biosensors’ overall effectiveness [14]. Numerous NMs are utilized in biosensor design, with a focus on their unique measurements and features. The NPs, as zero-dimensional (solid, hollow, and quantum dots (QDs)), one-dimensional (nanowires (NWs), nanotubes (NTs), and CNTs), two-dimensional (films, plates, and sheets), and three-dimensional (nanocomposites and polycrystals), provide valuable insights into the distinct NMs that are employed, highlighting their noteworthy contribution to augmenting the efficacy and responsiveness of biosensing systems. NMs can be developed using several methods and techniques [15], such as the “top-down” method and the “bottom-up” method. The approaches named top-down and bottom-up are used for synthesizing NMs and are schematically shown in Figure 4. This figure illustrates an easy-to-understand overview of several methods, showing how the NMs are prepared from specific elements to build big structures or developed from the macro-level to nanoscale sizes [16]. Several approaches are included in the bottom-up approach, which include the hydrothermal, CVD, pyrolysis, spinning, sol–gel, and other methods. The top-down approach incorporates a wide range of techniques, including thermal decomposition, sputtering, laser ablation, mechanical milling, and lithography. This extensive spectrum of methods highlights the intricate nature and adaptability of bottom-up and top-down approaches to NP development. Biosensor technology can undergo a radical change due to meticulous research, including CNTs, nanocomposites, NRs, NWs, and QDs. Understanding the fabrication and development of such NMs opens up opportunities for upgrading biosensors’ detection abilities. These NMs provide an amazing platform for modification and fine-tuning, permitting scientists to exactly fit them to the demands of various biosensing applications. Precise engineering provides opportunities to manage the selectivity and the overall effectiveness of biosensors, in addition to making it possible for them to attain higher levels of sensitivity.

### 2.1. Biosensors Based on Carbon Nanotubes

The first research on CNTs, referred to as buckytubes, was published by Sumio Ijima in 1991. Such structures are made of hollow carbon and have nanoscale diameters. They exhibit an appropriate geometry of carbon atoms, which are joined by sp2 bonds, which contribute to their strength and stiffness [17]. They have become the most researched family of NMs for the development of biosensors utilized in several health as well as scientific domains for diagnostic applications, and they also act as a superstructure to restrain biomolecules on their outermost surface.

A SWNT-oriented DNA sensor with excellent sensitivity and responsiveness was developed by Tang et al. in 2006 [18]. In 2013, Li et al. utilized semiconducting single-walled CNTs (s-SWCNT) to develop a biosensor that was capable of detecting dopamine at ambient temperature; the detection limit was extremely low at 10–18 mol/L [19]. Table 1 provides a summary of several CNT-based biosensors that utilize different analytes. To coordinate the physiological processes in the human body, neurotransmitters are crucial, particularly in facilitating intricate chemical messages between the brain networks of neuronal cells. Considerable advancement has been achieved in recent decades in the development of analytical approaches intended to measure neurotransmitter concentrations. Janssen et al. developed a CNT-based biosensor with a superior range of detection for the detection of bovine serum albumin (BSA) [20,21]. Florian et al. made significant advances in neurotransmitter monitoring by developing a novel fluorescent carbon nanotube-based sensor system [22]. Their research employed a systematic approach to modify the organic phase surrounding single-walled carbon nanotubes (SWCNTs), creating multiple sensor variants with distinct sensitivity and selectivity profiles specifically designed for catecholamine neurotransmitter detection. The study provided comprehensive insights into sensor functionality by establishing the complex relationships between DNA sequencing and SWCNT architecture. A key breakthrough was the sensor’s ability to distinguish between different catecholamine neurotransmitters and identify them, even in the presence of structurally similar interfering molecules. This enhanced discrimination capability represents a major improvement over existing methods, enabling more accurate and precise measurements in neurotransmitter monitoring applications. This development has profound implications since DNA-functionalized SWCNT-based sensors have the prospective to transform the scientific comprehension of neurotransmitter communication in intricate biological environments. The sensors possess enhanced sensitivity and selectivity, making them potentially useful instruments for interpreting the complex interactions between neurotransmitters across healthy and pathological environments.

These studies demonstrate that CNT-based biosensors provide exceptional conductivity and ultralow detection limits across diverse analytes due to their high aspect ratio, excellent conductivity, and favorable biomolecule immobilization. However, their performance is generally hindered by batch-to-batch synthesis variability and leads to challenges in achieving consistent functionalization, which restricts reproducibility and large-scale applications. In comparison with graphene, CNTs demonstrate better mechanical flexibility and impactful aspect ratios, which make them better suited for wearable and implantable biosensors. On the other hand, if we consider graphene, it exhibits more consistent reproducibility and planar structure due to its planar structure and facile surface chemistry. As a result, CNT shows better results in the field of flexible and wearable biosensors, whereas graphene and CNT-graphene hybrids tend to show better performance in reproducible large-scale biosensor fabrication.

### 2.2. Biosensors Based on Metal Oxides

NMs based on metal oxides possess the potential to improve biosensor responses and sensitivity due to their distinct chemical and physical properties at the nanoscale. The effective transfer of charge and transmission of signals can be facilitated by the one-dimensional structure offered by NWs and NRs. These NMs also have remarkable electrical, mechanical, and thermal characteristics, and each of them is capable of being used for improving the performance of biosensors. The precise amplification of signals and sequencing made possible by QDs, along with customizable optical characteristics, leads to an improved level of accuracy in biosensor devices. Opportunities for revolutionary breakthroughs regarding detection capacities become apparent as the fields of nanotechnologies and biosensor research come closer together. Target analyte detection with previously unheard-of levels of sensitivity, specificity, and efficiency is made possible by the meticulous processing and integration of these kinds of NMs into biosensor technologies. Through the utilization of the intrinsic benefits of nanoscale structures, scientists can expand the capabilities of biosensor technology and reshape its applications in numerous domains, including environmental monitoring, medical diagnosis, and beyond.

The oxides of cadmium (CdO), cobalt (Co_3_O_4_), copper (CuO), iron (Fe_2_O_3_), manganese (MnO_2_), nickel (NiO), tin (SnO_2_), titanium (TiO_2_), zinc (ZnO), and other elements have been employed in several industries over the last 20 years, owing to their broad spectrum of electrical, chemical, and physical characteristics. The most promising magnetic NMs with high electron transfer rates amongst the abovementioned oxides of metals are oxides of copper, iron, manganese, and zinc; consequently, they are used in the development of electrochemical biosensors [40,41].

#### 2.2.1. Biosensor Based on Oxides of Copper

Two of the copper oxides, CuO and Cu_2_O, are non-toxic NMs that are inexpensively and easily synthesized in large quantities. The process of synthesis can be modified more thoroughly to produce highly crystalline NPs with the appropriate dimensions, which can be used to create biosensors with exceptional specifications. Since copper oxides are p-type semiconductors, they are highly sought after for use in the production of sensors as well as rechargeable batteries, supercapacitors, solar cells, field-emitting devices, etc. [42,43]. The powerful oxidizing and bleaching material hydrogen peroxide (H_2_O_2_) is extensively used in organizations, residences, and biomedical settings. Furthermore, H_2_O_2_ is an important reactive oxygen species (ROS) that is involved in several processes that are both physiological and pathological. Its crucial role is highlighted by its association with a variety of human health issues, among which are diabetes, metabolic disorders, tumors, and other neurological diseases like Parkinson’s, Alzheimer’s, and Huntington’s chorea. For this reason, precise H_2_O_2_ monitoring is of utmost importance when it comes to scientific and practical uses in industry. It is essential to build H_2_O_2_ sensors that are quick, cheap, sensitive, and discriminating to meet this need. Nowadays, a multitude of sensor systems have surfaced to identify hydrogen peroxide. For the detection of H_2_O_2_, a carbon ionic liquid electrode was created using copper oxide NPs by Ping et al. The detection limit was continuously found to be between 1.0 μM and 2.5 mM, having a lower detection limit of 0.5 μM [44]. Palladium-copper oxide NPs were used by Dhara et al. to decorate reduced graphene oxide to create a biosensor determining the presence of glucose. The biosensor had a 30 nM lower detection limit [45,46].

An electrochemical sensor made from a CuO-graphene nanocomposite was developed by Z. Monsef Khoshhesab and detects acetaminophen, ascorbic acid, and caffeine simultaneously. The detection limit was 0.008, 0.011, and 0.010, respectively, while the linear spectrum of detection was from 0.025 to 5.3 μmol L^−1^ [47]. Comparably, Zhang et al. came up with NPs of CuO adorned with carbon spheres for the electrochemical measurement of glucose, achieving a sensitivity of 2981 μA mM^−1^ cm^−2^ [48]. The many CuNP-based electrochemical biosensors that have been in use recently are shown in Table 2. Cheng et al. presented an additional exemplary case of a sensor, which is based on paper, a colorimetric sensor that uses CuO hollow spheres to sense H_2_O_2_. Notable features associated with these mesoporous CuO hollow spheres include their significant volume of pores (0.56 cm^3^/g), large surface area (58.77 m^2^/g), available mesopores (5.8 nm), consistent diameter (~100 nm), and hollow shape. The CuO hollow spheres serve their purpose on inexpensive, reusable filtering paper testing strips by taking advantage of their characteristics. The resulting paper-based sensor works well in the 2.4–150 μM range for H_2_O_2_ detection. With substantial advantages for a range of applications, this novel strategy offers a viable path for effective and trustworthy H_2_O_2_ detection [49].

The above-mentioned reports state that CuO-based nanomaterials offer catalytic activity and low-cost fabrication, making them suitable for point-of-care biosensors. In comparison to ZnO and Fe_2_O_3_, they have low conductivity and stability issues, which limit their performance. But when combined with graphene, the hybrid CuO-graphene revokes these issues by combining catalytic efficiency with high electrical conductivity and durability. Hence, pure CuO yields low-cost rapid sensors, but the future will rely on CuO composites for stability in high-performance biomedical and environmental domains.

#### 2.2.2. Biosensor Based on Oxides of Iron

The iron oxides Fe_2_O_3_ and Fe_3_O_4_ have been widely used to synthesize several kinds of electrodes to develop a vast range of biosensors that are capable of detecting the molecules of organic matter, ions of heavy metals, and other substances. To develop a urea sensor, Kaushik et al. coated a glass plate with indium-tin oxide in 2009, before depositing a thin layer of Fe_3_O_4_ NPs/chitosan. In the case of urea, the detection limit was 0.5 mg/dL, while the concentration range was 5–100 mg/dL [58]. Using an Ag-Fe_2_O_3_-graphene oxide magnetic nanocomposite, Li et al. created a nitrite sensor in 2015 that had a linear spectrum ranging from 0.5 μM–0.72 mM and a lower limit of detection of 0.17 μM [59]. Fe_2_O_3_/graphene NPs were utilized by Lee et al. in 2016 for the creation of an electrochemical sensor that could detect Zn^2+^, Cd^2+^, and Pb^2+^ metal ions. For Cd^2+^, Zn^2+^, and Pb^2+^, these were identified throughout a linear range of 1–100 μg L^−1^, with 0.08 μg L^−1^, 0.11 μg L^−1^, and 0.07 μg L^−1^ serving as the lowest levels of detection [60]. Table 3 also shows the electrochemical biosensors based on iron oxide NPs that have been utilized recently in several applications. Iron oxide (Fe_2_O_3_) is a type of transition metal oxide that is not only widely used but has also gained attention in many different fields due to its remarkable electrochemical properties, outstanding biocompatibility, affluence, and availability. Amongst all of their applications, α-Fe_2_O_3_ NPs, or α-Fe_2_O_3_ NPs, have attracted a lot of interest as a spectacular regulating agent. This is explained by the fact that iron oxides have the innate ability to experience in situ electrochemical oxidation or reduction because of their changeable valence state, which causes heterogeneous redox reactions. Research has demonstrated how important nanostructured α-Fe_2_O_3_ morphologies are for magnetic, optical, electrochemical, and photocatalytic characteristics. The intriguing topic of how shape affects electrochemical sensing, especially with tiny biomolecules, remains to be explored. To detect serotonin, Ran et al. created an electrochemical sensor with a lower detection limit of 80 nM and a linear concentration range of 0.5–100 mM [61]. The sensor was made using Fe_2_O_3_ and bromocresol green embedded in the chitosan matrix. The morphology-dependent electrochemical sensing characteristics of iron oxide-graphene oxide nanohybrids for uric acid and dopamine were revealed by Cai et al. [62], in light of this requirement. Using a simple hydrothermal approach mediated by meta-ions, the study produced iron oxide NPs (Fe_2_O_3_ NPs) with discal, rhombic, and cubic morphologies. The study team combined graphene oxide (GO) nanosheets with the remarkable electrocatalytic activity of discal Fe_2_O_3_ NPs (d-Fe_2_O_3_) in an attempt to improve the electrochemical sensing capabilities. The oxidation of dopamine (DA) and uric acid (UA) was achieved with exceptional electrocatalytic efficiency due to the synergistic interaction between discal Fe_2_O_3_ NPs and GO. Importantly, in the concentration ranges of 10–100 μM and 0.02–10 μM, respectively, this partnership enabled linear electrochemical responses for both UA and DA. The remarkably low limits of detection (LOD) of 2.5 nM for UA and 3.2 nM for DA further highlighted the method’s sensitivity. The d-Fe_2_O_3_/GO nanohybrids, in particular, demonstrated excellent selectivity and repeatability, opening up exciting new possibilities for sophisticated electrochemical sensing applications.

#### 2.2.3. Biosensor Based on Oxides of Manganese

Manganese oxide (MnO) has special characteristics, such as its ability to exist in a variety of oxidation states, because of which it is becoming a potent tool for the development of biosensors. These states enable vital electron transfer activities in biosensing mechanisms. Because of its adaptability, MnO can interact with a wide range of biomolecules. MnO is perfect for electrochemical biosensors due to its exceptional catalytic activity. Its capacity to mediate electron transport between biomolecules and electrode surfaces can lead to sensitive and efficient detection. Its tunable behavior is noteworthy. By adjusting the characteristics, scientists may create biosensors that can detect a wide range of analytes. MnO’s usefulness in biosensing is further increased by its high compatibility with a variety of biomolecules. Other advantages are provided by MnO nanostructures such as NPs, NWs, and nanosheets. Due to their large surface-to-volume ratio, these nanostructures offer enough room for the immobilization of biomolecules. Stringer signals and increased detection sensitivity result from this. The increased surface area of MnO nanostructures facilitates signal amplification, enabling the detection of even minute concentrations of the target analytes. It is now widely known that MnO has the potential to be used in the creation of biosensors. Its distinct physiochemical characteristics make it very advantageous for enzyme-based biosensors. Its capacity to transfer electrons straight between electrodes and enzymes, doing away with the requirement for extra mediator molecules and improving the stability of the biosensor overall, is one of its main advantages. By combining these qualities, MnO nanostructures are being incorporated into biosensing technology that offers remarkable sensitivity and low detection limits, opening the door to the development of many industries, such as food security, environmental monitoring, and medical diagnosis. Furthermore, enzymes bound to MnO surfaces maintain their bioactivity due to their biocompatibility. This results in consistent and dependable performance in applications involving biosensing. MnO’s versatility extends beyond its role in enzyme-based sensors. The diverse range of oxidation states it can exhibit allows interaction with several varieties of biomolecules. Manganese oxides, including MnO, MnO_2_, and Mn_3_O_4_, are attracting significant interest for their potential in biosensor development. These oxides offer a compelling combination of characteristics that make them ideal for various applications of sensing. First of all, they are economical and ecologically beneficial. MnO, in contrast to certain other materials, is easily accessible, non-toxic, and needs low-cost synthesis methods. Furthermore, they exhibit remarkable efficacy in alkaline conditions. They are ideal for a variety of biosensing applications due to their high energy density and alkaline activity [72,73,74,75,76,77]. Their varied dimensionality is yet another important benefit. MnO comes in a variety of forms, ranging from 3-D structures to 0-D nanoparticles. Higher-dimensional structures, such as 1-D and 3-D forms, have a substantially bigger surface area than 0-D NPs, as Table 4 illustrates. The sensitivity and performance of the biosensor are eventually enhanced by the increased number of potential reaction sites that result from this increased surface area. Overall, the combination of their eco-friendly nature, cost-effectiveness, strong performance in alkaline environments, and diverse dimensionality makes manganese oxides a promising material for the next generation of biosensors.

Despite their catalytic activity and multiple oxidation states, which enable electron transfer in biosensing, pristine MnO_2_ demonstrates very low conductivity and selectivity, limiting its applicability to upgrading the performance of nanocomposites that can be introduced as MnO_2_-Carbon/metal hybrids. MnO-based biosensors have become a cost-effective, catalytically versatile material that is important in composite biosensors.

#### 2.2.4. Biosensor Based on Oxides of Zinc

Zinc oxide (ZnO) stands out as an optimal material for developing biosensors owing to its chemical stability, eco-friendly nature, and high isoelectric point (IEP). This IEP property allows ZnO to effectively capture target molecules like DNA, enzymes, and proteins through electrostatic interactions [87,88]. ZnO NPs offer even greater versatility due to their diverse dimensionality, ranging from 0-D (zero-dimensional) to 3-D (three-dimensional). Each dimension provides unique advantages for biosensor design, allowing for tailored performance and functionality to address specific detection requirements. These characteristics collectively enhance the sensitivity, selectivity, and overall effectiveness of ZnO-based biosensors. ZnO NPs come in various dimensions (0-D to 3-D), each offering distinct advantages for biosensor design as 0-D (Zero-Dimensional): High surface area allows for efficient biomolecule attachment, ideal for capturing trace analytes in ultra-sensitive detection. 1-D (One-Dimensional): Signal transmission is enhanced by swift and persistent electron transfer channels, which result in sensors that are more precise and receptive. 2-D (Two-Dimensional): Specified planes are useful for multi-analyte biosensing because they allow for the simultaneous identification of multiple analytes. 3-D (Three-Dimensional): Increased overall surface area allows for more biomolecule attachment, boosting sensitivity for capturing target molecules. This tailored approach using ZnO nanostructures paves the way for advancements in biosensing across various fields, such as medical diagnostics (early disease detection through highly sensitive and specific biosensors), environmental monitoring (real-time detection of pollutants in soil, water, and air), and food safety (sensitive detection of pathogens and contaminants in food items). By harnessing the unique properties of each dimension, researchers can create powerful and versatile biosensors for numerous applications. ZnO’s remarkable sensing properties and biocompatibility make it a highly versatile material for biosensor development. Researchers have successfully employed ZnO-based biosensors to detect Naproxen. Tashkhourian et al. (2014) achieved a detection limit of 2.3 × 10^−7^ M using a carbon paste electrode modified by ZnO NPs and multi-walled CNTs [89]. Bashami et al. (2015) developed ZnO-coated carbon electrodes for detecting para-nitrophenol, having a limit of 0.02 μM [90]. Fang et al. (2016) have shown 3-D ZnO sensors for glucose detection with a limit of 0.02 mM [91]. These examples showcase ZnO’s potential for biosensing applications beyond traditional targets. With ongoing research, ZnO-based sensors can become more cost-effective, sensitive, and selective, paving the way for advancements in environmental monitoring, medical diagnostics, and food safety. A critical need exists for sensitive, affordable, and portable biosensors capable of detecting pesticides in several settings. This technology would be invaluable for applications in environmental monitoring, agriculture, and food packaging. Researchers have made significant strides in this area. A recent study by Fallatah et al. (2022) provided a promising ZnO nanostructure-based biosensor for pesticide detection [92]. This sensor was developed by immobilizing the acetylcholinesterase (AChE) enzyme on ZnO NPs directly grown on a flexible porous surface. An exceptional performance is exhibited by ZnO biosensors developed on carbon cloth. They achieved a detection limit from 0.5 nM to 5 μM for organophosphate (OP) pesticides, showcasing high sensitivity and improved stability. This research highlights the immense potential of ZnO-based biosensors for practical pesticide detection. Further development in this area could lead to the creation of cost-effective and user-friendly tools for ensuring food safety, monitoring agricultural practices, and safeguarding the environment.

ZnO nanostructures stand out for their wide bandgap, high isoelectric point, and strong photocatalytic activity, which leads to efficient biomolecule/enzyme immobilization and biosensing applications. ZnO generally possesses lower electron transfer rates when compared to MnO and CuO, which may limit sensitivity in certain electrochemical applications, but this can be enhanced by hybrid ZnO-graphene or ZnO-polymer composites. ZnO is stable in aqueous and physiological environments, which empowers its robustness. ZnO is suitable for low-cost, eco-friendly biosensors, whereas MnO and CuO perform well in catalytic-driven applications.

### 2.3. Biosensors Based on Nanorods

NRs, measuring 1–100 nanometers in diameter, are a promising material for biosensor development [93,94]. They can be synthesized from several materials like graphene, semiconductors, and metal oxides. NRs have demonstrated significant potential for the detection of a vast range of biological targets, including carbohydrates, nucleic acids, and metal ions. Researchers are actively exploring the use of NRs in biosensors. Sun et al. (2013) employed graphene oxide and graphene NRs to create a biosensor for detecting bovine IgG [95]. Further, in 2017, Hahn et al. designed a field-effect transistor (FET) biosensor utilizing zinc oxide NRs for the detection of phosphate [96]. This technology was further refined by Zhu et al. (2018), who achieved high-sensitivity glucose monitoring with a detection limit of 1 μM using a similar FET biosensor [97]. Beyond this, Liu et al. (2019) developed a fluorescence resonance energy transfer biosensor for lead ion detection utilizing carbon dots and gold NRs [98]. Additionally, Bagyalakshmi et al. developed a ZnO NR-based enzymatic glucose biosensor on a chitosan film, achieving a linear detection range from 10 μM to 40 μM for glucose concentrations in 2020 [99]. Volatile organic compounds (VOCs) present in exhaled breath hold promise as biomarkers for various diseases. Acetone and isopropanol are linked to diabetes and lung cancer, respectively. Kankan Swargiary et al. (2022) introduced a novel optical fiber sensor for selective VOC detection [100]. This sensor utilizes a zinc oxide (ZnO) coating to target isopropanol, a potential biomarker for diabetes. A core silica fiber (CSF) sandwiched between two single-mode fibers (SMFs) is used in the sensor’s design to create a structured SMF-CSF-SMF architecture. Multimode interference (MMI) is made possible by this arrangement in the CSF, which increases light interaction at the fiber-sensing medium interface and ultimately improves detection sensitivity. To further enhance sensor performance, the researchers used simulations to optimize the CSF length for optimal coupling efficiency at the output. The researchers used a low-temperature hydrothermal technique to grow ZnO NRs directly onto the coreless silica fiber surface to increase sensitivity. This novel method preserved the structural integrity of the fiber while producing a strong sensing platform. Following that, the ZnO-coated fiber sensor was evaluated using different isopropanol (IPA) vapor concentrations (20%, 40%, 60%, 80%, and 100%). The sensor displayed impressive performance, accurately detecting isopropanol and achieving a high sensitivity of 0.053 nm/% IPA vapor. This demonstrates the sensor’s ability to not only distinguish but also quantify isopropanol’s presence. These results highlight its capability for non-invasive diabetes monitoring and pave the way for broader applications in the medical field.

### 2.4. Biosensors Based on Nanowires

NWs are considered powerful tools for developing next-generation biosensors. These tiny, solid structures, typically made from semiconducting metal oxides, carbon, or even metal NTs, possess remarkable properties despite their minuscule size. Unlike bulk materials, NWs exhibit exceptional characteristics that span the mechanical, thermal, chemical, optical, and electronic realms. These exclusive characteristics make them extremely attractive for building biosensors with significantly improved sensing as well as detection limits for a vast range of analytes [101,102]. As shown in Table 5, various metal NW-based biosensors have been successfully developed to detect diverse targets. Researchers are actively exploring the potential of NWs in biosensing, as evidenced by several advancements.

A poly-silicon NW biosensor was created by Hakim et al. in 2012 to identify inflammatory biomarkers with high sensitivity and a broad concentration range binding capability [103]. Label-free optical silicon NW biosensors were later introduced by Irrera et al. in 2018 for the detection of C-reactive protein in human serum, having an excellent detection range of 10^−2^–100 μg/mL [104]. Priolo et al. (2018) employed silicon NW optical biosensors for detecting human blood genomes with high sensitivity [105]. This development demonstrates the enormous potential of NWs to transform biosensor technology and pave the way for more sensitive biosensors with a greater variety of uses. The potential of silicon NW FETs (Si-NW FETs) to detect genetic markers associated with cancer was investigated in 2021 by Ivanov et al. [106]. By utilizing the benefits of Si-NW FETs, such as their compatibility with current mass production techniques, this integration of established technologies holds promise for advancements in cancer detection. The foundation of their approach is Si-NW sensors that are specifically designed to detect genetic markers linked to cancer; the Si-NW FET configuration is anticipated to provide remarkable precision and sensitivity in identifying these markers due to the exclusive electrical characteristics of NWs. The tiny size, as well as the high surface-to-volume ratio of silicon NWs, enables highly sensitive molecular interactions. The researchers sought to create a reliable approach for detecting biomolecular signals particular to cancer by taking advantage of these innate benefits. The creation of next-generation biosensors with important ramifications for early cancer detection is made possible by this research.

**Table 5:** Recent advances in metal nanowire-based biosensors: target analytes, nanowire configurations, transduction methods, and analytical performance.

Sensor Type	Synthesis Method	Mechanism	Target Analyte	Detection Range	Ref
DNA-functionalized Au	Chemical vapor transport	SERS	Uranyl Ion	10^−7^–10^−12^ M (1 pM)	[107]
Silver	Commercial source	Piezoresistive Sensing	Strain	80–0% Strain (0.2%)	[108]
Pt and Pt Ox	E-beam fabrication	Chemical Resistance	Hydrogen	1000–0.5 ppm (100 ppm)	[109]
Nickel-gold layered	Electrochemical deposition	Electrochemical	Glucose	2–0.0025 mM (0.1 μM)	[110]
Nickel	Electrochemical deposition	Chemical Resistance	Hydrogen	20–0.01 mM (0.8 μM)	[111]
Palladium-PAN composites	Electrospinning	Chemical Resistance	Hydrogen	4–0.0001% (1 ppm)	[112]
Core–shell Pd@Ag	LPNE/GRR	Chemical Resistance	Hydrogen	900–100 ppm (100 ppm)	[113]
Gold	Oriented attachment	Chemical Resistance	DNA	1–0.001 nM (1 pM)	[114]
Copper phosphide	Hydrothermal synthesis	Electrochemical	Glucose	1–0.005 mM (0.32 μM)	[115]
Graphene-gold hybrids	Hydrothermal synthesis	Cyclic Voltammetry	Tulobuterol	7.6–0.076 μmol/L (0.01361) μmol/L	[116]
AuPt polydopamine	Hydrothermal synthesis	Voltammetry	Pesticides	1000–0.5 ng/L (0.185 ng/L)	[117]
Au-decorated CoS_2_	Hydrothermal/Lithography	Chemiluminescence	Hydrogen Peroxide	100–1 μM (0.03 μM)	[118]
Gold	Nanoimprint lithography	Square wave Voltammetry	CRP	220–5 fg/mL (2.25 fg/mL)	[119]
Jagged Pt Ni	Solvothermal method	Electrochemical	Caffeic Acid	0.75–600 μM (0.05 μM)	[120]

### 2.5. Biosensors Based on Quantum Dots

QDs are tiny semiconductor crystals, typically measuring 2–10 nanometers in diameter [121]. Notably, their size dictates their color as follows: QDs between 5 and 6 nm appear red or orange, while smaller ones (2–3 nm) emit green or blue light. These properties, along with their shape and structure, influence their overall characteristics. One common synthesis method for QDs involves breaking down larger carbon materials, such as CNTs or graphite, into these minuscule nanodots (top-down approach) [122]. Owing to their unique optical characteristics, QDs have emerged as promising substitutes for fluorescent molecules (fluorophores) in biosensor design. These biosensors can detect an extensive range of biological molecules, like macromolecules and organic compounds.

Table 6 provides a detailed look at various QD-based biosensors used for detecting different analytes. The exclusive properties of QDs make them highly versatile for biosensing applications. Different kinds of QDs have been investigated in research, which identifies a variety of analytes. The literature reports that Zhang et al. used nitrogen-doped carbon QDs (N-CQDs) to successfully detect mercury (II) ions at a low detection limit (0.23 μM) in 2013 [123]. Similarly, Saini et al. (2017) presented a thiol-functionalized fluorescent CQD chemosensor to detect arsenite with a wide range of 5–100 ppb [124]. Additionally, Amjadi et al. (2017) utilized nitrogen and sulfur co-doped CQDs to create a chemiluminescence sensor for indomethacin determination, attaining a concentration range of 0.1–1.5 mg L^−1^ and 65 μg L^−1^ detection limit [125]. In another study, Wang et al. (2018) incorporated graphene QDs (GQDs) into a photoelectrochemical aptasensor to detect zeatin with a broad range [126]. A novel approach by Savas and Altintas (2019) explores an electrochemical sensor utilizing graphene quantum dots (GQD) as nanozymes for Yersinia enterocolitica detection [127]. The GQD-based immunosensor achieved exceptional sensitivity across a wide range of concentrations, detecting as few as 5 colony-forming units (CFU) per mL in milk and 30 CFU/mL in serum samples. Beyond Yersinia enterocolitica detection, this innovative electrochemical approach using GQDs holds immense potential for revolutionizing pathogen diagnosis in clinical and food safety settings. The ability to eliminate pre-sample treatment steps paves the way for a more efficient, rapid, and cost-effective method for detecting various pathogenic bacteria in diverse samples. This GQD-based immunosensor is capable of transforming the field of infectious disease diagnosis, offering significant benefits for both clinical and food safety applications.

These reports highlight the QDs strength in fluorescence tunability, multiplexing capability, and high sensitivity across chemical, pharmaceutical, and pathogenic targets. Cadmium-based QDs have toxicity concerns and scalability issues, which restrict their application areas. In comparison to CNTs and metal oxides, QDs have an excellent approach in optical biosensing, but biocompatibility and reproducibility seem to be issues. Carbon and graphene-based QDs, hybrid QD graphene/polymer composites, offer good sensitivity along with improved safety and stability for clinical and food safety.

## 3. Applications of NMs Used for Biosensor Development

The application of diverse NMs in developing biosensors is presented in Table 7. This is intended to deliver a detailed overview of the work conducted over the past two decades in this field.

The unique physiochemical properties of nanomaterials play a critical role in defining the performance of biosensors. Table 8 reflects a comparative analysis of key nanomaterials. Each of these nanomaterials brings specific advantages depending on the biosensing applications.

CNTs are preferred for electrochemical and flexible wearable sensors due to their high conductivity and adaptability to various geometries. Graphene offers a balance between conductivity and flexibility, making it suitable for FET-based and stretchable biosensors. Metal oxides are ideal for environmentally robust biosensors where chemical or thermal resistance is necessary. Quantum dots are optimal for highly sensitive optical detection platforms where fluorescence or colorimetric responses are required. Material selection must be aligned with the target analyte, intended environment, and sensor type.

## 4. Adversities and Upcoming Patterns in Nanobiosensors

The anticipated expansion of the global population to 8.5 billion by 2030 presents formidable healthcare challenges, particularly regarding diagnostic accessibility and affordability. This demographic trend threatens to exacerbate healthcare disparities, especially in developing regions like India, necessitating the development of efficient Point-of-Care Technologies (POCT). The emergence of nanotechnology has introduced promising materials such as CNTs, graphene, and QDs for diagnostic applications, though their widespread implementation faces significant regulatory and safety hurdles. Modern POCT applications extend beyond conventional medical diagnostics to encompass diverse biological sample analyses, environmental monitoring, and the detection of pathogens. The convergence of AI and cyber-physical systems has accelerated the development of sophisticated nanobiosensor platforms. These systems require multidisciplinary advances in scientific and engineering domains to boost their selectivity and sensitivity across various applications, from in vitro diagnosis to drug delivery systems. Nanobiosensors offer healthcare professionals and researchers powerful tools for detecting specific biomarkers, nucleic acids, proteins, and enzymes. While traditional assays exist, their limitations in processing time, multiple analyte requirements, and accuracy highlight the urgent need for rapid, reliable, multiplexed screening methods. The advancement of these technologies promises to address critical healthcare accessibility challenges while providing more accurate and efficient diagnostic solutions.

## 5. Conclusions

NM-enhanced biosensors, which combine biological recognition with physicochemical transduction, represent an innovative class of analytical devices. These sophisticated platforms demonstrate exceptional capabilities in detecting diverse analytes, ranging from carbohydrates and metal ions to gases, amino acids, and disease-specific biomarkers. The incorporation of NMs in biosensor design capitalizes on their unique properties, enhancing detection sensitivity, selectivity, reproducibility, and stability. These improvements stem from the NMs’ superior electron transport properties, high surface-area-to-volume ratios, and enhanced electrochemical characteristics. The future trajectory of nanobiosensors encompasses advances in automation, integration, and miniaturization, accelerated by convergent technologies, including IoT, machine learning, cloud computing, and AI. Nanobiosensors, emerging from the synthesis of sensor engineering, biotechnology, and nanotechnology, are revolutionizing point-of-care diagnostics. These devices show remarkable potential for transforming healthcare delivery and expanding into diverse industrial applications through real-time, in situ monitoring capabilities. Despite notable progress, unmet challenges persist, including scalable fabrication, long-term stability, and standardized performance protocols. Future trends point to AI-integrated biosensing, flexible/wearable electronics, IoT-enabled real-time diagnostics, and sustainable nanomaterial synthesis. Key research gaps involve multiplexed detection, improved biocompatibility, and cost-effective mass production. Actionable steps include advancing reproducible nanofabrication, establishing international standards, fostering interdisciplinary collaboration, and exploring eco-friendly materials. Addressing these priorities will accelerate translation from prototypes to impactful, real-world diagnostic and monitoring solutions. The evolution of NM-based biosensors marks a crucial technological advancement, promising to revolutionize diagnostic approaches across multiple fields.

## Figures and Tables

**Table 1:** Carbon nanotube-based biosensors: a comparative overview of analytes, bioreceptors, and detection methods.

Type of Sensors	Device Configuration	Synthesis Methods	Target Analyte	Detection Limit	Ref
CNTs	Amperometric	Dielectrophoresis	Streptavidin	100.0 aM	[22]
		Dielectrophoresis	HER2 antibody	10.0 fM	[22]
		Drop–Coat (Paper Filter)	Formaldehyde	0.016 ppm	[23]
	Chemoresistive	Direct Contact Printing	H5N1 DNA	2.0 pM	[24]
		Drop Coating	PSA	1.18 ng/mL	[25]
		Dieletrophoresis	H_2_	10 ppm	[26]
		Dieletrophoresis	NO_2_	0.5–20 ppm	[27]
		Drop Coat	NH_3_	100 ppb	[28]
		Drop Coat	N-nitroso dialkylamine	1 ppb	[29]
		Immersed in a solution of Carbon Nanotubes	H_2_	0.89 ppm	[30]
		Spray Deposition	NH_3_	10 ppm	[31]
		Spray Deposition	CO_2_	600 ppm	[31]
		Spray Deposition	CO	3 ppm	[31]
		Spray Deposition	Ethanol	17 ppm	[31]
		CCVD	Toulene	50 ppm	[32]
		Dieletrophoresis	Tetrahydrocannabinol	0.163 ng	[33]
		Drop Coat	NH_3_	2 ppm	[34]
		Drop Coat	NO_2_	2 ppm	[34]
	Spin Coat	FET	DNA	880 ng/L	[35]
	FET	Dielectrophoresis	Cortisol	50 nm	[36]
		Dielectrophoresis	NPY	500 pm	[36]
		Dielectrophoresis	DHEAS	10 nM	[36]
		OTS Masking	Aspergillus niger	-	[37]
		CCVD	N_2_+ ion	Single ion	[24]
		Immersed in CNT Solution	DNA	60 aM	[38]
		Immersed in CNT Solution	Micro vesicle	1 ppml	[38]
		OTS Masking	Aquaporin-4	1 ng	[39]

**Table 2:** Summary of CuO nanoparticle-based electrochemical biosensors: analyte targets, electrode platforms, transduction strategies, and analytical performance metrics.

Copper Oxide System	Target Analyte	Detection Method	Detection Limit	Ref
CuO-Graphene/CPE	Acetaminophen	Differential Pulse Voltammetry	0.006 μM	[49]
CuO-Graphene/CPE	Caffeine	Differential Pulse Voltammetry	0.011 μM	[49]
Cu_x_O/ERGO	Dopamine	Electrochemical	11.0 nM	[50]
CuO/g-C_3_N_4_ composites	Dopamine	Electrochemical	1 × 10^−10^ mol/L	[51]
Cu_2_O-TiNTs	Eugenol	CV	1.32 μM	[52]
Cu_2_O-BSA NPs	Glucose	CV	0.41 μM	[53]
CuO/GO	Glucose	Electrochemical	0.67 μM	[54]
CuO-rGO	Glucose	Amperometry	0.12 μM	[55]
Cu_2_O-rGO/GCE	H_2_O_2_	Amperometry	21.8 μM	[56]
CuO NPs/CCE	Tyrosine	Amperometry	160.0 nM	[57]

**Table 3:** Electrochemical biosensors based on iron oxide nanoparticles: a comparative overview of target analytes, sensor configurations, transduction mechanisms, and performance metrics.

Iron Oxide Composites	Target Molecule	Detection Technique	Detection Limit	Ref
Fe_3_O_4_/rGO composite	Ascorbic Acid	DPV	0.44 μM	[63]
Fe_3_O_4_ NPs with CB/GCE	Bisphenol A	DPV	0.032 nM	[64]
Fe_3_O_4_/rGO composite	Dopamine	Amperometric	5.0 nM	[65]
rGO/Fe_3_O_4_/Gelatin CPE	Glucose	CV	0.022 μM	[66]
P Py-chitosan-Iron oxide	Glucose	Electrochemical	225.0 μM	[67]
PEG-Fe_3_O_4_on GE	L-Dopa	DPV	9.6 nM	[68]
Ag@Fe_2_O_3_/SPCE	Nitrate	Amperometric	30.0 μM	[69]
Fe_2_O_3_/GCE	Pyrocatechol	Chronoamperometry	-	[70]
Fe_3_O_4-_modified	Tyrosine	DPV	50.0 nM	[71]
Fe_2_O_3_ NPs	Uric Acid	Electrochemical	2.4 nM	[62]

**Table 4:** Manganese oxide nanoparticle-based electrochemical biosensors: overview of analyte targets, electrode configurations, sensing strategies, and analytical performance metrics.

Dimensions	Improved Electrode	Detection Method	Sample	Detection Range	Ref
0-D	MnO_2_ NPs on Ta MnO_2_ NSPs-GNR/SPCE	CV & Amperometric	Milk Honey	1–2 μM 0.1–1.4 mM	[78,79]
0-D	MnO_2_ NSPs-GNR composites MnO_2_ NPs-Polythiophene/GCE	Electrochemical	Honey Human serum	0.1–1.4 mM 0.04–9 μM	[79,80]
1-D	Au/MnO_2_ NNDs/SPCE MnO_2_ NRs-HBCs/SPE M13-E4@MnO_2_NWs	Amperometric CV and Chrono amp. Electrochemical	Blood Plasma Blood Serum, Peach Juice	0.3–5.1 μM 28–93 μg/ML 5 μM–2 nM	[81,82,83]
2-D	MWCNT-MnO_2_/rGO/Au MnO_2_ NSs/GCE Lucigenin/MnO_2_ NSs/GCE	CV Electrochemical ECL	Serum SP2/0 cells Human Serum	0.1–100 μM 2–10 μM 10–2000 nM	[72,84,85]
3-D	MnO_2_ nanomesh/GCE MnO_2_ NFs/N-rGO MnO_2_ NFs/3D-RGO/Ni	Electrochemical	Blood, Urine Human Serum Pork	0.2–10 mM 6–10 μM 17–962 nM	[75,86,87]

**Table 6:** Summary of quantum dot-based biosensors: analyte targets, QD compositions, sensor formats, detection techniques, and analytical performance.

Quantum Dot Material	Detection Method	Matrix	Target Molecule	Detection Range	Ref
CdS MOF structure	ECL	Human serum	Carcinoembryonic antigen	-	[128]
α-FeOOH with CdS/Ag	ECL	-	17β-estradiol	0.01–10.0 pg/mL	[129]
MoS_2_ coupled with GQDs	Electrochemical	Wine Matrices	Caffeic acid	0.38–100.0 μM	[130]
CdTe	Fluorometric	Bio fluids	Dopamine	0.5–10.0 μM	[131]
Polymer-CdTe/CdS	Fluorometric	Human fluids	Glucose	0.2–5.0 mM	[132]
MoS_2_ integrated CdTe	Fluorometric	Milk	Tetracycline	0.1–1 μM	[133]
Nickel-doped CdTe	Fluorometric	Plasma	Pyrazinamide	2.0–100.0 μM	[134]
ZnCdS MIP coating	Fluorometric	Vitamin C formulations	Ascorbic acid	1.0–500.0 μM	[135]
CdTeS coated with SiO_2_	Image analysis	Serum	Folic acid	5.0–80.0 μM	[136]
CdTe	PET	Synthetic media	ds DNA	0.0874–20 μg/mL	[137]

**Table 7:** List of various nanomaterials used in biosensor development over the last two decades.

Nanomaterial	Transducer	Target Analyte	Detectable Amount	Ref
Gold nanobipyramids	SPR	Aflatoxin B1	0.4 nanomolar	[138]
Gold NPs	Electrochemical	Uranyl ions	0.3 μg/L	[139]
Gold NPs	Fluorescence	Lead ions	16.7 nanomolar	[140]
Gold/CdS QDs on titanate NTs	Electrochemical	Cholesterol	0.012 micromolar	[141]
Gold NP-MoS_2_-rGO	SAW	Carcinoembryonic antigen	0.084 ng/mL	[142]
Gold/rGO	Electrochemical	miRNA-122	1.73 picomolar	[143]
Silver NPs	Colorimetric	Hydrogen peroxide	0.032 micromolar	[144]
Silver/palladium NPs	Electrochemical	Ractopamine	1.52 pg/mL	[145]
Silver@carbon QDs-rGO	Electrochemical	Dopamine	0.59 nanomolar	[146]
Platinum NPs	Voltammetric	Adrenaline	2.93 × 10^−4^ mol/L	[147]
Platinum-iron oxide@carbon	Amperometric	Sarcosine	0.43 micromolar	[148]
Copper/reduced graphene oxide-black phosphorus	Electrochemical	Glucose	11 micromolar	[149]
Nickel/copper metal–organic framework	Field-effect transistor	Glucose	0.51 micromolar	[150]
Cobalt oxide nanocubes	Electrochemical chip	Glutamate	10 micromolar	[151]
Manganese oxide-Mn_3_O_4_@reduced graphene oxide	Impedimetric	Hydrogen peroxide	0.1 micromolar	[152]
Zinc oxide NRs	Field-effect transistor	Phosphate	0.5 millimolar	[153]
GQDs	Electrochemical	Copper ions	1.34 nanomolar	[154]
CdS/CdTe/ZnS QDs	Fluorescence	L-ascorbic acid	1.8 × 10^−9^ molar	[155]
Gold NPs@polydopamine@CuInZnS QDs	Electrochemiluminescence	P53 gene	0.03 nmol/L	[156]
Silicon NWs	Field-effect transistor	Virus of Dengue	2 femtomolar	[157]
Gold NPs@polydopamine@CuInZnS QDs	Electrochemiluminescence	P53 gene	0.03 nmol/L	[156]
Silicon NWs	Field-effect transistor	Virus of Dengue	2 femtomolar	[157]
Graphene-gold NRs	Amperometric	NADH	6 micromolar	[158]
Cobalt oxide-carbon nanotube/titanium dioxide	Photoelectrochemical	Glucose	0.16 micromolar	[159]
Graphene QDs-multi-walled CNTs	Electrochemical	Dopamine	0.87 nanomolar	[160]
PAMAM dendrimer	Optical fiber	Dengue virus envelope protein	19.53 nm/nM	[161]

**Table 8:** Comparative analysis of nanomaterials in biosensors.

Nanomaterial	Electrical Conductivity	Sensitivity	Mechanical Flexibility	Environmental Stability	Applications	Ref
CNTs	Excellent	High	Very high	Good	Neurotransmitter detection, strain sensor	[162,163,164,165]
Graphene	Superior	High	Excellent	Moderate	pH sensor, FET-based detector	[166,167,168]
Metal Oxides (ZnO, CuO, Fe_2_O_3_)	Moderate	Moderate	Moderate	Excellent	Electrochemical detection of H_2_O_2_, glucose, urea	[169]
Quantum Dots (QDs)	Moderate	Extremely high	Low	Low	Optical biosensor, cancer biomarker	[170]

## Data Availability

No new data were created or analyzed in this study.

## Abbreviations

The following abbreviations are used in this manuscript

CCVD	Catalytic Chemical Vapor Deposition
CNT	Carbon Nanotubes
QD	Quantum Dots
NW	Nanowires
NR	Nanorods
NP	Nanoparticles
NM	Nanomaterials
NT	Nanotubes
CVD	Chemical Vapor Deposition
FET	Field Effect Transistor
CV	Cyclic Voltammetry
DPV	Differential Pulse Voltammetry
